# Pulsed Photothermal Heterogeneous Catalysis

**DOI:** 10.1021/acscatal.2c05435

**Published:** 2023-02-22

**Authors:** Andrea Baldi, Sven H. C. Askes

**Affiliations:** Department of Physics and Astronomy, Vrije Universiteit Amsterdam, De Boelelaan 1081, 1081 HV Amsterdam, Netherlands

**Keywords:** photothermal catalysis, pulsed light, plasmonics, microkinetic simulations, out-of-equilibrium, nonsteady state

## Abstract

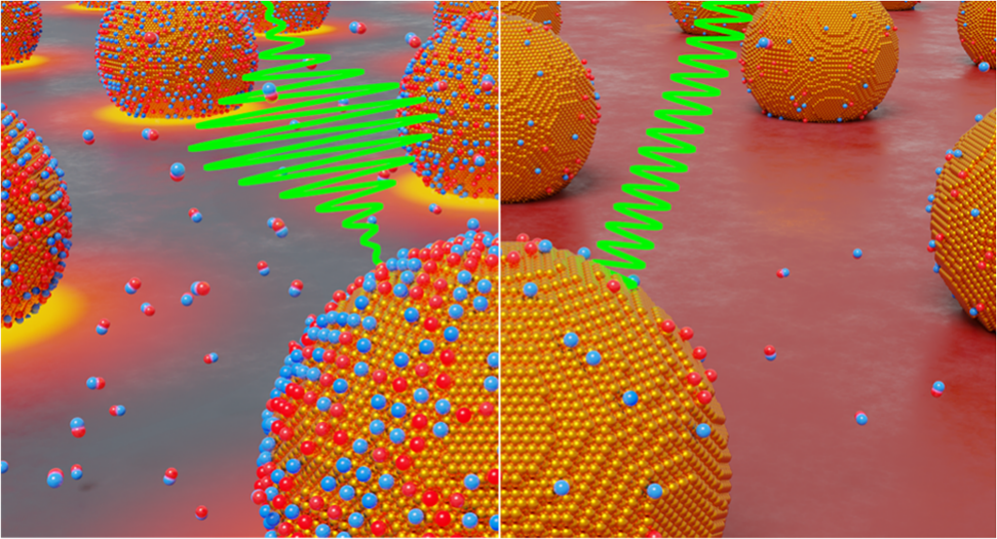

Anthropogenic climate change urgently calls for the greening and intensification of the chemical industry. Most chemical reactors make use of catalysts to increase their conversion yields, but their operation at steady-state temperatures limits their rate, selectivity, and energy efficiency. Here, we show how to break such a steady-state paradigm using ultrashort light pulses and photothermal nanoparticle arrays to modulate the temperature of catalytic sites at timescales typical of chemical processes. Using heat dissipation and time-dependent microkinetic modeling for a number of catalytic landscapes, we numerically demonstrate that pulsed photothermal catalysis can result in a favorable, dynamic mode of operation with higher energy efficiency, higher catalyst activity than for any steady-state temperature, reactor operation at room temperature, resilience against catalyst poisons, and access to adsorbed reagent distributions that are normally out of reach. Our work identifies the key experimental parameters controlling reaction rates in pulsed heterogeneous catalysis and provides specific recommendations to explore its potential in real experiments, paving the way to a more energy-efficient and process-intensive operation of catalytic reactors.

## Introduction

Catalysts are the workhorses of industrial chemistry, accounting for 85% of chemical products, such as artificial fertilizers and precursors for plastics.^1^ Although catalysts greatly speed up processes by reducing reaction barriers, chemical reactors remain energy-intensive and require high temperatures and pressures to reach appreciable conversion rates. One of the limiting factors for intensifying, greening, and improving the selectivity of catalytic processes with conventional chemical reactors is the operation under steady-state conditions, with no temporal control over temperature and reagent surface coverages. In contrast, operation under non-steady-state conditions, by rapid variation of temperature, can lead to higher reactor output, higher energy efficiency, and differences in product selectivity, which has been theoretically examined for more than 50 years,^2,3^ and more recently experimentally demonstrated.^4−8^ The idea behind such a “pulsed” mode of operation is that the catalyst surface is rapidly switched from low temperatures to high temperatures, thereby performing catalysis with a high rate and with surface coverages that are ordinarily not associated with steady-state high temperatures.

So far, pulsed heating has been implemented using electrical microreactors with a thermal modulation longer than 25 μs,^4−6^ resulting in reaction rates for CO oxidation on Pt 40 times higher than under steady-state heating at the same average power. However, electrical heating suffers from a high thermal inertia of the system, leading to significantly slower heating and cooling compared to the ps–ns timescales of elementary reaction steps and adsorption and desorption of surface species. This temporal mismatch can result in a situation where adsorbate surface coverages still respond quickly enough to reach steady-state conditions during the temperature increase and decrease, thereby limiting the potential impact of pulsed heating. To go beyond slow electrical heat modulation, the use of pulsed light coupled to photothermal materials offers ultimate spatial–temporal control of heating and can result in efficient light-to-heat conversion within hundreds of fs and fast cooldown periods in the ns range.^9,10^ Although photothermal heating of catalyst nanoparticles with CW light has been implemented since more than 30 years,^11^ the use of pulsed light was only recently considered.^7,12^

Plasmonic nanoparticles (NPs) are attractive photothermal materials, thanks to their large and wavelength-tunable absorption cross sections, their ability to efficiently convert light to heat in subwavelength volumes,^13−15^ and their catalytically active surfaces.^16,17^ Furthermore, under fs–ps pulsed illumination, the heat is particularly well concentrated around the nanoparticles for up to several ns,^18^ leading to extreme thermal gradients and exponentially enhanced reaction rates otherwise inaccessible under continuous wave (CW) illumination or any other steady-state heating method. The result is that pulsed optical heating of plasmonic nanoparticles offers a unique possibility to apply short and intense thermal bursts at the catalyst surface, without the need to heat up the entire reactor. To the best of our knowledge, only one study to date has directly compared plasmonic photothermal catalysis under CW and pulsed conditions, under the same wavelength and the same intensity.^12^ In this study, pulsed (5.5 ns) illumination caused a 50× higher CO_2_ hydrogenation rate with an Au@ZnO nanocatalyst compared to the same intensity CW illumination.

Despite the predicted advantages and because experimental data are still scarce, a clear understanding is lacking of how pulsed plasmonic heating can be efficiently used to drive photothermal catalysis. Especially, the coupling of spatiotemporal heat dynamics to chemical kinetics has been unexplored. In this work, we use microkinetic modeling with time-dependent optical-heat input to explore the conditions in which pulsed optical heating of plasmonic nanoparticle arrays can outcompete steady-state heating. We specifically focus on variables regarding array design (interparticle distance), chemical kinetics (activation energy), and adsorbate binding strengths.

In the first part of the paper, we shortly describe the essence of plasmonic photothermal heating and the differences between pulsed and CW illumination. We then introduce the optical heating and microkinetic chemical modeling approach, discuss the results for a second-order catalytic reaction, and additionally explore scenarios of reagent poisoning and product poisoning. Finally, we present recommendations for the experimental realization of pulsed photothermal catalysis. Overall, we prove that a pulsed mode of operation grants access to a vastly different kinetic landscape and can lead to energy efficiencies that are greatly enhanced compared to steady-state heating.

## Plasmonic Photothermal Catalysis in a Nutshell

Metal nanostructures are highly polarizable by light, thanks to the collective oscillation of their free electrons at the frequency of the incoming electric field, also known as localized surface plasmon resonance (LSPR). This phenomenon leads to outstanding absorption cross sections that can exceed the physical cross-sectional area of the nanostructure, as well as to focusing of light in subwavelength volumes.^19,20^ Tuning the shape, size, and material of the nanoparticle grants control over wavelength response, whether the light is scattered (radiative decay) or absorbed (nonradiative decay) and whether the light is absorbed in the entire volume or at a subnanoparticle level.^13^

**Figure 1:**
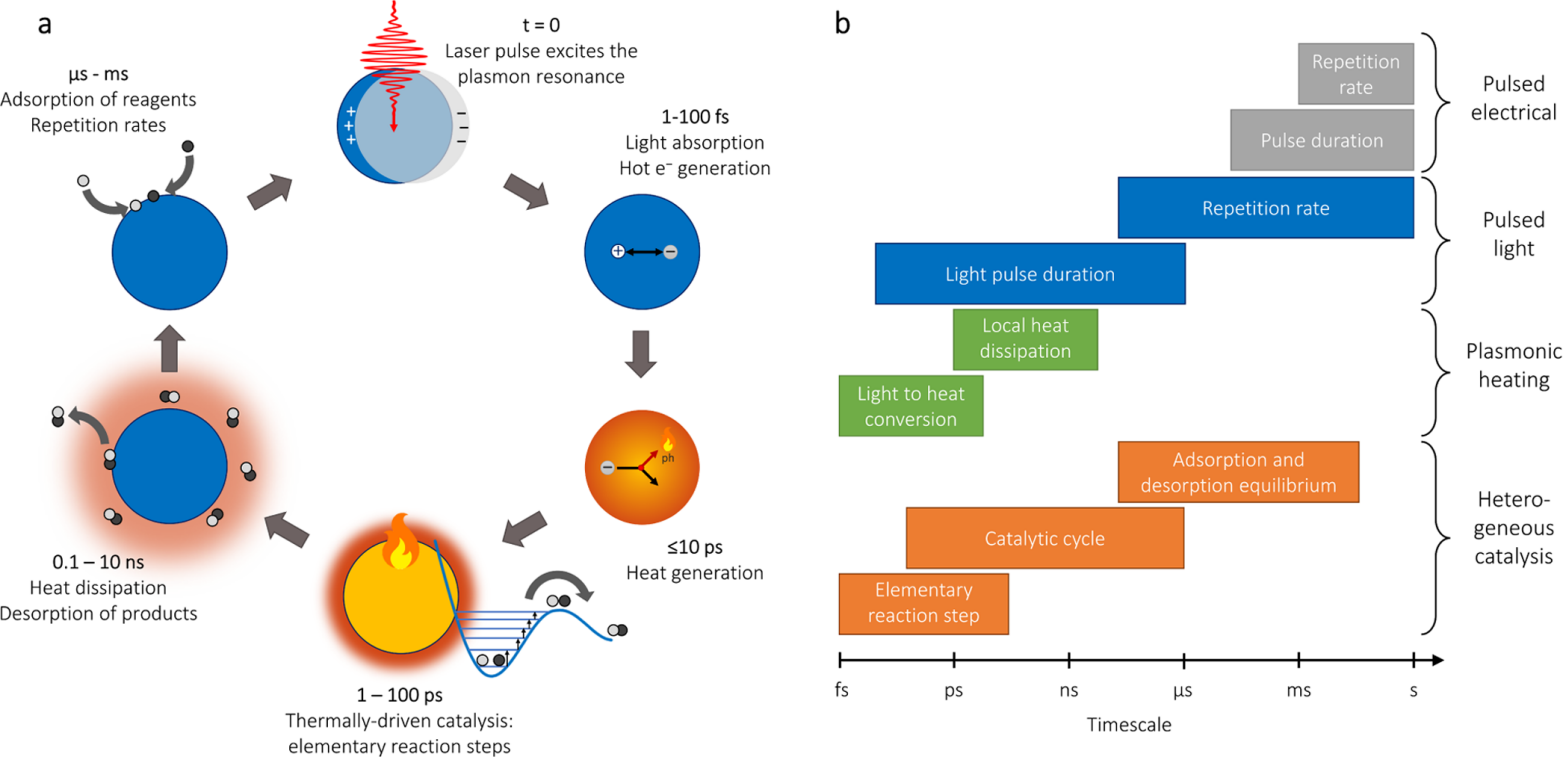
Schematic representation of (a) the dynamics of pulsed plasmonic photothermal catalysis and (b) the typical timescales involved.^25,26^

In Figure 1, we describe the important timescales for a plasmonic particle that is photothermally heated to drive a heterogeneous catalytic reaction. In the case of nonradiative decay, dephasing of the resonance within a time range of 1–100 fs leads to absorption of the energy and the promotion of electrons to higher energy levels (Figure 1a). The resulting “hot” electrons and holes quickly lose their excess energy through electron–electron scattering (<100 fs) and electron–phonon coupling (≤10 ps for Au and Ag),^21^ heating up the lattice of the particle. Meanwhile, the hot lattice vibrationally excites chemisorbed reactants, which are usually strongly coupled both electronically and vibrationally, on a sub-ps timescale.^22−24^ Sufficient vibrational excitation initiates the elementary reactions steps of bond breaking and forming to convert a reagent into product, within a fs to 100 ps timeframe.^25,26^ Next, the adsorbed product desorbs from the hot surface, and the plasmonic nanostructure cools back down to ambient temperature by heat transfer to the local surroundings within a time range of 0.1–10 ns, depending strongly on the effective thermal conductivity of the medium. Finally, in the dark pulse-to-pulse time, new reagents adsorb until the cycle is reinitiated with the next flash of light. Crucially, plasmonic heat generation and dissipation under pulsed conditions temporally overlap with the typical rates of elementary reaction steps and catalytic cycles, which is in stark contrast with what is possible with pulsed electrical heating (Figure 1b). Further, the available repetition rate of laser sources (down to 12 ns pulse-to-pulse time) temporally overlaps with the timescale of both catalytic cycles and adsorption/desorption equilibria. Thus, tuning the repetition rate and light pulse duration of plasmonic heating offers the unique possibility to directly influence thermocatalytic processes.

Alternative nonthermal chemical reaction pathways induced by optical near-fields, hot electrons and holes, and various energy transfer mechanisms are also possible.^27,28^ However, considering that the efficiencies for chemical reactions driven by these processes are currently far from industrially irrelevant (<1%),^27,29^ we here focus exclusively on maximizing the photothermal mechanism.

## Pulsed vs Continuous Wave Illumination

Distinct thermal regimes are accessible by varying the light source from CW to pulsed, with great implications for chemical reactions. In general, under CW excitation, heat is very poorly confined around the nanoparticle because of the long time between incoming photons compared to the rate of heat dissipation.^30^ In contrast, under pulsed photothermal heating, one can access orders of magnitude higher temperatures and thermal gradients for the same average intensity and apply short bursts of intense heat directly to the catalyst surface.^18,31^ The magnitudes involved can be appreciated by calculating the temperature increase (Δ*T*) of a single spherical Au nanoparticle on glass in air under 532 nm CW and pulsed illumination.

Under CW conditions, plasmonic heating depends on the nanoparticle’s absorption cross section (σ_abs_ in m^2^), the light intensity (*I* in W/m^2^), the nanoparticle’s radius (*r* in m), and the average thermal conductivity of the medium (κ in W/(K m)) according to^30,31^
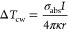
1Thus, shining an unfocused 532 nm laser with 1 mW power and 1 mm diameter (0.13 W/cm^2^) on a single 50 nm diameter Au nanoparticle (σ_abs_ ≈ 2 × 10^–14^ m^2^) on a glass substrate in air (κ_effective_ ≈ (κ_glass_ + κ_air_)/2 ≈ 0.4 W/(K m)) elevates its temperature by only 0.0002 K, which is a negligible effect for chemical reactions.

In contrast, if a fs-pulsed laser would be used with the same wavelength and average intensity, at 1000 Hz repetition rate (*f* in s^–1^), the maximum temperature elevation (Δ*T*_p_^max^), before any heat transfer to the surroundings, during each ultrashort light-flash can be calculated from the pulse energy density (*I*/*f* = 0.13 mJ/cm^2^), absorption cross section, mass of the particle (*m* = 1.26 × 10^–18^ kg), and the heat capacity of the material under constant pressure (*C*_p_ = 128 J/(kg K))
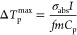
2For the same Au nanoparticle on glass, the maximum temperature elevation would be Δ*T*_p_^max^ = 157.5 K, corresponding to a transient temperature increases 6 orders of magnitude higher than its steady-state equivalent under CW light with the same average intensity. In practice, when the heat flux to the surroundings competes with the internal lattice heating, Δ*T*_p_^max^ is lower, as is the case for metals with relatively slow electron–phonon coupling (τ_e–ph_ > 0.2 ps), for small nanoparticles (*r* < 20 nm), and for a high interfacial heat conductivity (low Kapitza resistance).^18^ Since there are no photons in between pulses, the nanoparticle lattice cools down again to ambient temperature, typically following a stretched exponential with timescales between 0.1 and 10 ns^32^
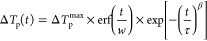
3Here, the time constant (τ) and the stretching parameter (β) are independent of laser intensity but only depend on the nanoparticle size, thermal conductivity of the surrounding medium, and the interfacial heat conductivity.^18^

**Figure 2:**
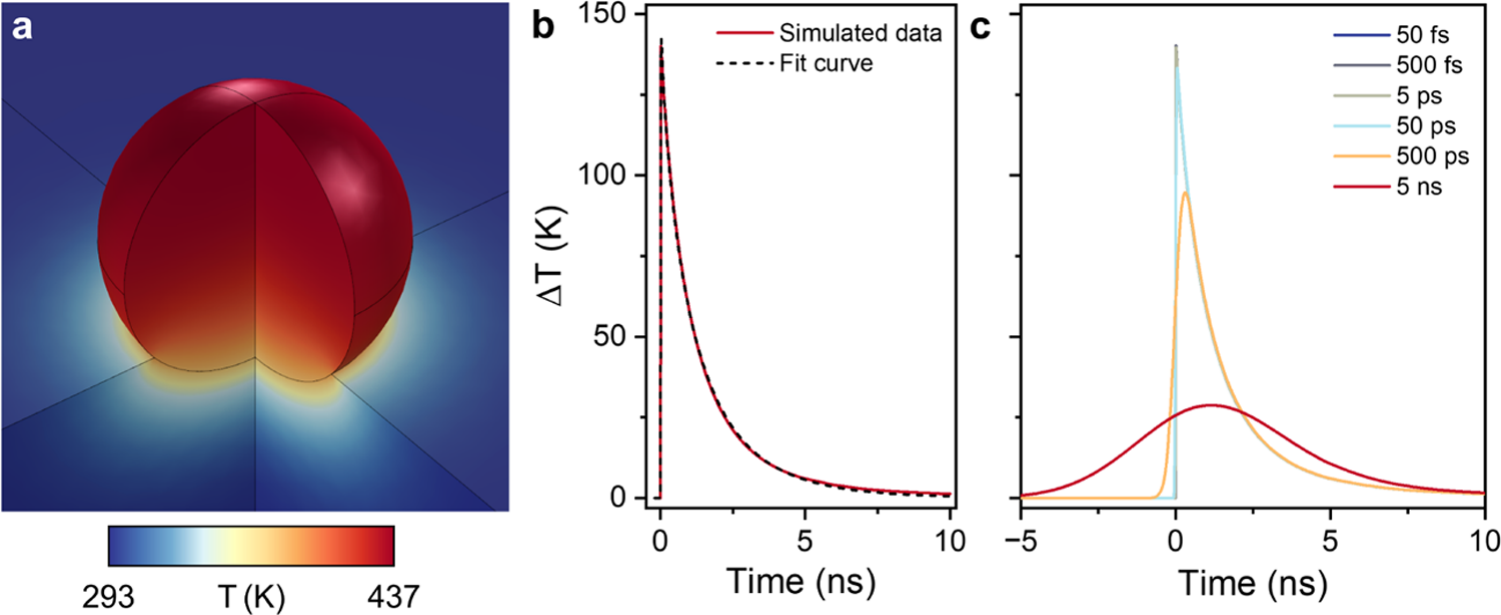
Finite element method simulations of a 50 nm diameter Au nanosphere supported on SiO_2_ in air under 50 fs pulsed excitation (0.13 mJ/cm^2^). (a) Spatial lattice temperature distribution calculated using a two-temperature model 14 ps after the optical pulse. (b) Decay of the averaged surface temperature of the nanoparticle (red solid line) and fitted curve according to eq 3 (black dashed line). (c) Temporal decay of the averaged surface temperature of the nanoparticle for 50 fs, 500 fs, 5 ps, 50 ps, 500 ps, and 5 ns pulsed excitations (FWHM). The 50 fs, 500 fs, and 5 ps decay curves are closely overlapping.

To obtain realistic heating and heat dissipation dynamics for a single 50 nm diameter Au NP on glass, as input for our chemical kinetics modeling, we performed finite element method (FEM) simulations with an experimentally verified three-dimensional two-temperature model under pulsed excitation (50 fs FWHM pulse width), as shown in Figure 2.^9,10,21^ The bottom 10 nm of the Au NP was embedded in the glass support to approximate a higher degree of contact area, as is often observed in TEM imaging.^33^ The results showed that the top of the nanoparticle achieves a maximum surface temperature of 154 K after 14 ps, close to the theoretical Δ*T*_p_^max^ value (eq 2), with a small temperature gradient (<10 K) toward the bottom, which is in contact with the glass support (Figure 2a). The heat dissipation to the surroundings is well fitted with eq 3 (τ = 1.07 ns; β = 0.77) and is complete within 10 ns (Figure 2b). The fitted stretching parameter β was in line with experimental values obtained for aqueous nanoparticle dispersions (0.5–0.7).^10,32^ Furthermore, the results confirmed that the maximum surface temperature is linear with light intensity, while the exponential decay parameters τ and β are minimally affected (Figure S1).

The laser pulse width was also varied between 50 fs and 5 ns (FWHM), while keeping the same average intensity (Figure 2c). These data showed that Δ*T*_p_^max^ and the decay parameters were essentially the same up to 50 ps excitation. For the longer pulse widths of 500 ps and 5 ns, the peak temperatures diminish to 68 and 21% of Δ*T*_p_^max^, respectively, because the thermal efflux is greater than the optical heating influx. These substantially lower peak temperatures are detrimental for thermal catalysis because even though illumination with the shortest pulses decreases the timeframe of heating, during the short bursts of intense heat, the catalytic activity increases (approximately) exponentially. For example, when using these peak temperatures to calculate reaction rate constants according to the Arrhenius equation (100 kJ/mol activation energy), the peak rate constant for 50 fs illumination is 26× greater than for 500 ps and 15,000× greater than 5 ns. Therefore, because pulse widths below 50 ps maximize the highest obtainable Δ*T*_p_^max^ and therefore the chemical reaction rate, such short pulses are most attractive for driving photothermal catalysis. In the microkinetic model used throughout this paper, we use the fitted thermal decay curve that we obtained from our simulated data with 50 fs excitation.

When moving from single, isolated nanoparticles to nanoparticle assemblies, as is typical for photothermal heterogeneous catalysis, the local temperature of each nanoparticle is additionally raised by nearby particles. This temperature increase is also known as collective heating (Δ*T*_ext_)^30,31^ and allows high temperatures to be reached under moderate light intensities, even under CW excitation. In this work we focus on infinite square lattice arrays of spherical nanoparticles, for which Baffou et al. have shown that Δ*T*_ext_ depends inversely on the lattice unit cell area (*a*) and beam diameter (*D*) according to^30^
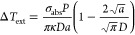
4All our simulations are done under the assumption that collective heating contributions are time-independent for both CW and pulsed illumination. In other words, we are assuming that the collective heat stored in the particle array and its surroundings does not dissipate significantly in between pulses at the repetition rates used in this work (≤1 ms). This approximation can be justified by estimating the upper and lower boundaries for the effective thermal diffusivity *D* of the array at the air–glass interface, defined as^34^
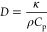
5where *D* is 0.4 mm^2^/s for glass and 19 mm^2^/s for air. For a 1 mm beam diameter (0.79 mm^2^), the heat dissipation time constant is calculated by^35^
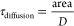
6where τ_diffusion_ has a value between 41 ms (pure air) and 2 s (pure glass). Crucially, this time constant is much longer than the slowest repetition rate explored in this study, and we can therefore safely assume that the time dependence of collective heating effects can be neglected.

## Chemical Kinetics under Pulsed and CW Illumination

**Figure 3:**
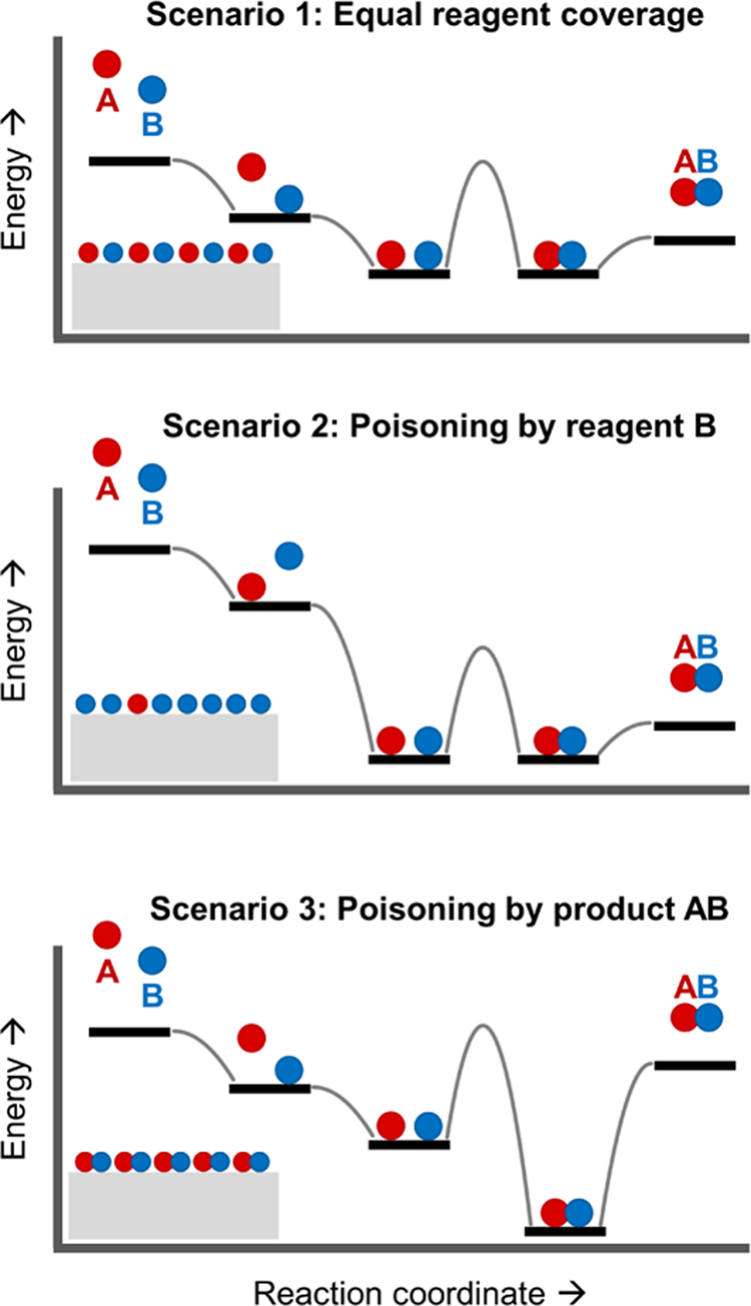
Qualitative energy diagrams and reaction schemes of the three catalysis scenarios considered in this work. In scenario 2, the surface is poisoned by reagent B, which leads to uneven reagent coverages and an inefficient reaction. In scenario 3, the surface is poisoned by product AB, which leads to low reagent surface coverages and an inefficient reaction.

To assess the implications of pulsed illumination for heterogeneous catalysis, we consider a generic gas-phase heterogeneous catalysis reaction with second-order kinetics (A + B → AB) and compute the formation rate of the product AB under CW and pulsed illumination using microkinetic modeling with a Langmuir–Hinshelwood-type mechanism (Figure 3, scenario 1).^1^ In this model, reagents A and B adsorb onto an empty catalyst site (*) with the same adsorption energy (*E*_ads_^A^ = *E*_ads_^B^ = −50 kJ/mol; no activation barrier) and both with a sticking probability (*S*_ads_) of 10%, according to
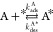
7
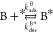
8
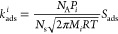
9where *N*_A_ is Avogadro’s constant, *P*_*i*_ is the partial pressure of reagent *i* (*P*_A_ = *P*_B_ = 0.5 atm), *N*_s_ is the number of sites (here, set as the approximate number of surface atoms, i.e., 1 × 10^19^ sites/m^2^), *M*_*i*_ is the molar mass of reagent *i* (kg/mol), and *R* is the gas constant. The molar masses were chosen to be slightly different (*M*_A_ = 0.028 kg/mol and *M*_b_ = 0.032 kg/mol) to prevent the overlap of surface coverage data. Desorption for all species occurs according to Arrhenius-type kinetics that depend on the desorption energy (*E*_des_^i^ = −*E*_ads_^i^)
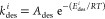
10In our model, pulsed illumination makes adsorption and desorption rates time-dependent by substituting *T* with *T*(*t*) in eqs 9 and 10.

After adsorption, A* and B* react to form AB* according to
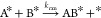
11
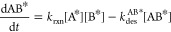
12Here, *k*_rxn_ depends on temperature, reaction activation energy (*E*_a_ in kJ/mol), and the pre-exponential factor (A, set to 10^14^ s^–1^ as a typical value for unimolecular surface reactions).^36^ The reaction rate constant (*k*_rxn_) depends only on temperature, and it is therefore either constant under CW illumination (eq 13) or strongly time-dependent under pulsed illumination (eq 14)
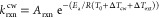
13
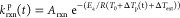
14As the last step of the catalytic cycle, the desorption of AB* (eq 15) is here set to be favorable when compared to the activation energy (*E*_des_^AB*^ = 20 kJ/mol), which allows neglecting the reverse catalytic reaction.
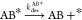
15For the three generic scenarios that we explore first, *E*_des_ and *E*_a_ are assumed to be independent of the surface coverage. To obtain the time-dependent production rate of AB, the system of differential equations governing [A*], [B*], [*], [AB*], and [AB] was numerically solved (details are given in the Supporting Information).

**Figure 4:**
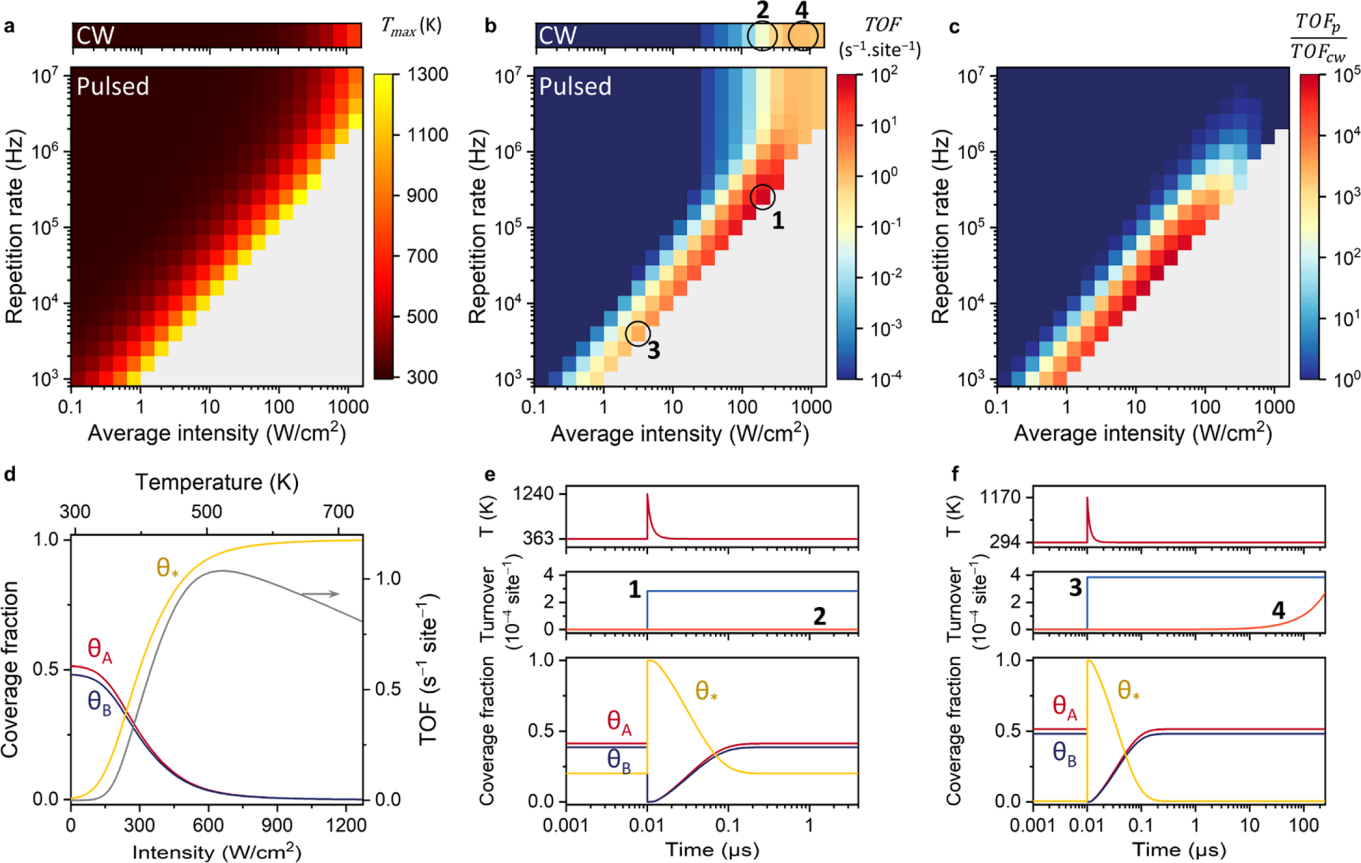
Microkinetic modeling under CW and pulsed illumination for a Au NP array with 600 nm pitch. (a) Maximum surface temperature of individual Au NPs under CW illumination (top) and under pulsed illumination as a function of average light intensity and pulse repetition rate (bottom). (b) Reaction TOF under CW illumination (top) and under pulsed illumination (bottom) calculated by solving the system of time-dependent differential equations specified in the Supporting Information. (c) Ratio of TOFs for pulsed and CW illumination. The experimental conditions labeled from 1 to 4 in panel (b) correspond to the blue and orange turnover data in panels (e) and (f). (d) Steady-state coverage fractions of A*, B*, and empty sites and the reaction TOF (gray, right axis) as a function of CW illumination intensity (bottom axis) or temperature (top axis). (e) Nanoparticle surface temperature (top), reaction turnover (middle), and coverage fractions of A*, B*, and empty sites (bottom) for a single pulse period at 200 W/cm^2^ and 250 kHz. The optical pulse is centered at 10 ns. The reaction turnover with the same intensity CW illumination is shown in orange. (f) Same as in panel (e) but for pulsed illumination at 3.2 W/cm^2^ and 4 kHz and CW illumination at 200 W/cm^2^.

As performance metric, we directly compared the turnover frequency (TOF) of AB (s^–1^ site^–1^) between pulsed and CW conditions for repetition rates of 1 kHz to 10 MHz and for optical powers between 1 mW and 10 W in a 1 mm diameter beam (0.13–1270 W/cm^2^ intensity), which reflects what is currently experimentally attainable with ultrafast pulsed lasers. For pulsed conditions, this TOF was calculated by integrating the production of desorbed AB for a single pulse period and multiplying by the repetition rate; this quantity can thus be regarded as a time-averaged TOF that is directly comparable with steady-state heating.
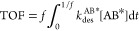
16As a starting point, we investigated the kinetics for a Au nanoparticle array on glass with 600 nm pitch, 100 kJ/mol reaction activation energy, and 50 fs pulse duration (FWHM), see Figure 4. Under CW illumination, such an array heats up by only 0.35 K per W/cm^2^ (Figure 4a). For pulsed illumination, transient temperatures can easily exceed the melting point of Au, where photoablation of nanospheres is expected to become prominent.^37−40^ For this reason, data points were omitted for transient nanoparticle temperatures exceeding 1300 K (occurring for >0.8 mJ/cm^2^ dose). Note that, while this critical temperature is valid for nanospheres, lower critical temperatures are expected for more intricate nanoparticle shapes, which will reshape into nanospheres.^39^

When comparing the TOF under CW and pulsed conditions (Figure 4b,c), close to the melting region of Au, a range of pulse parameters can be distinguished where pulsed illumination produces up to 5 orders more product than for CW, at the same intensity. For these conditions, the short bursts of extreme chemical productivity compensate for the dark time in between pulses, when the nanoparticle is cold. In general, decreasing the repetition rate at the same intensity increases the turnover, as few high-intensity heat bursts lead to more product than many low-intensity heat bursts. Following the same rationale, increasing the intensity at the same repetition rate increases the turnover as well. For the dark-blue regions in Figure 4b, the low energy per pulse results in a low peak temperature and the low productivity per pulse, thereby providing no advantage over CW conditions. For these parameters, the production during pulses does not contribute significantly to the overall rate.

Further inspecting these data indicated that pulsed illumination can outcompete CW illumination in terms of absolute catalyst activity. Pulsed illumination resulted in a 66× higher TOF than the highest TOF possible with CW at 2.5× less intensity or 1050× higher TOF when compared at the same intensity (Figure 4e, marks 1 and 2 in Figure 4b). This result can be understood as pulsed illumination at ambient temperature exploits the combination of high surface coverage and extremely high reaction rates, which are mutually exclusive under steady-state heating. To clarify this point, Figure 4d shows the surface coverages and the reaction rate under CW illumination, which are equivalent to conventional thermocatalysis at the same catalyst temperature. While the reaction rate increases exponentially (eq 13), the surface coverages decrease, thereby suppressing the reaction rate at higher temperatures and resulting in an optimum rate. Because of the exponential temperature dependence of the rate, however, such optimum in steady-state conditions is reached at very low surface coverages and therefore at relatively low conversion rates. This compromise does not apply to pulsed illumination, where the catalyst surface at the start of the pulse is cold and fully covered. In this way, pulsed illumination can substantially increase the maximally obtainable TOF of photothermal catalysts by orders of magnitude.

Additionally, pulsed illumination can lead to a higher energy efficiency than CW, intended as the number of product molecules per photon absorbed. It is in fact possible to find low-intensity pulsed conditions with a comparable TOF as high-intensity CW. For example, a combination of 3.2 W/cm^2^ and 4 kHz already achieves a higher TOF than the highest TOF possible with CW at 507 W/cm^2^ (Figure 4f, marks 3 and 4 in Figure 4b). In other words, with 160× fewer photons, a higher TOF is achieved. In both of these cases, pulsed illumination completely depletes the catalyst surface and produces AB in short bursts of high activity, resulting in a stepwise catalytic turnover in time (Figure 4e,f), which is in contrast to the slow accumulation of product under CW. In between pulses, the surface cools back down within 10 ns to ambient temperature, thereby allowing accumulation of adsorbed reactants before the next pulse arrives.

In the regime of high intensity and high repetition rate (Figure 4b, upper right corner), pulsed and CW illumination are very comparable. This similarity can be attributed to a combination of collective heating and reduced surface coverages. First, a high collective temperature results in a high reaction rate during the dark time between pulses, which lowers the relative impact of the pulse. Second, this same collective heating lowers the damage ceiling for pulses so that the melting temperature of Au is reached with a less pulse energy density. This decreases the catalytic impact of the pulse compared to collective heating. Finally, a higher steady-state temperature results in higher desorption rates of A* and B* (Figure 4d) up to the point where the surface is nearly empty and catalysis occurs with a diminished rate.

Overall, when particles are driven to temperatures close to their photoablation limit, pulsed light can result in drastically higher catalytic productivity and energy efficiency. These results highlight that choosing the right experimental parameters is critical for exploiting pulsed illumination in heterogeneous catalysis.

## Particle Density and Collective Heating

**Figure 5:**
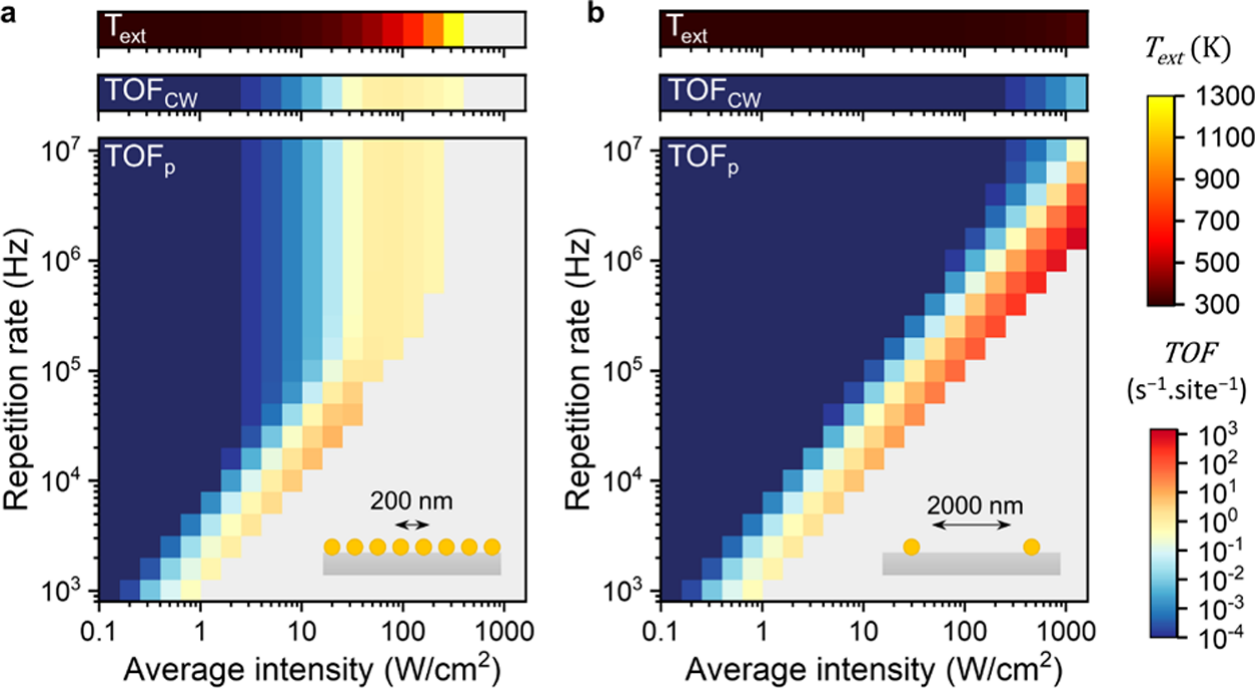
Role of collective heat in pulsed photothermal catalysis. Reaction TOF as a function of light intensity and pulse repetition rate for a Au NP array with (a) 200 nm pitch and (b) 2000 nm pitch. The collective heating temperature and TOF under CW illumination as a function of light intensity are shown in the top panels.

In the previous calculations, we used an interparticle distance of 600 nm. However, increasing the particle density increases the relative contribution of collective heating (eq 4 and Figure S2), which raises the question of how interparticle distance and collective heating influence pulsed photothermal catalysis. To answer this question, we consider two extreme situations. In the first case, a pitch of 200 nm was chosen, for which 50% of the light is absorbed, resulting in high collective heating temperatures of 3.1 K per W/cm^2^ (Figure 5a, top panel). Under these conditions, the melting temperature of Au is reached at a light intensity of 320 W/cm^2^. In the second case, a very sparse array was chosen with a pitch of 2000 nm (0.5% light absorption), resulting in an almost negligible collective heating of 0.033 K per W/cm^2^ (Figure 5b, top panel).

For the dense array and for repetition rates above 100 kHz, the catalyst activity is identical to that of CW owing to the dominance of collective heating, with the maximum TOF reached at 80 W/cm^2^. In short, the high activity in the dark time between pulses dwarfs the catalytic activity during pulses. Further, due to copious collective heating, a greater portion of the conditions exceeds the melting temperature of gold and desorption of reagents takes place at a lower intensity. These two factors prevent a high TOF to be reached under pulsed conditions. Therefore, the globally best obtained TOF (at 20 W/cm^2^ and 25 kHz) was only 7× the highest TOF possible with CW compared to 66× for 600 nm pitch (at 202 W/cm^2^ and 250 kHz).

For the sparse array with a pitch of 2000 nm, the situation is reversed. Even at the highest calculated intensity (1270 W/cm^2^), CW illumination only heats up the array’s particles by 42 K, leading to a negligible TOF (Figure 5b, CW panel). In contrast, under pulsed illumination, the nanoparticle produces 10^5^× more (at 1270 W/cm^2^ and 1.6 MHz) than CW and 560× more than what is maximally possible under steady-state heating (Figure 4d). Such productivity is in fact 1 and 2 orders of magnitude higher than for 600 nm and 200 nm pitch, respectively. These observations are fully explained by the near-absence of collective heating, which would otherwise deplete the surface coverage of reagents at such high-intensity conditions.

Taken together, these results highlight the profound effect of collective heating on the available parameter space for pulsed photothermal catalysis.

**Figure 6:**
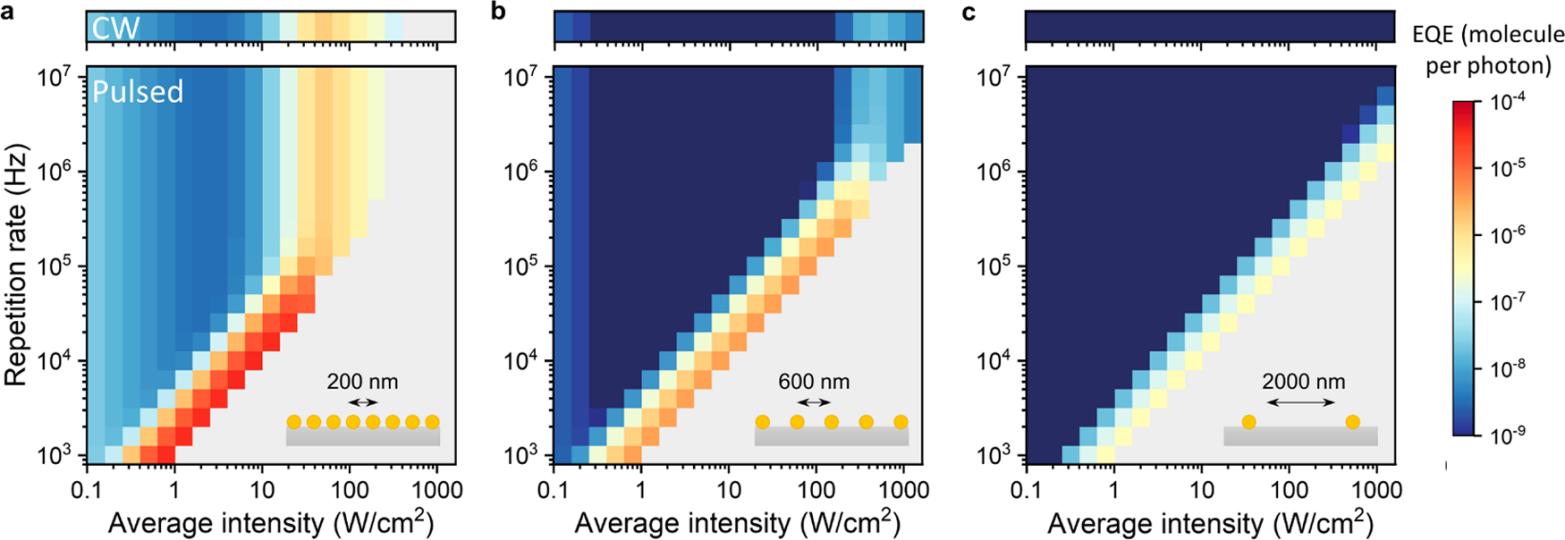
External quantum efficiency (EQE), expressed as product molecules per incident photon, as a function of light intensity and pulse repetition rate for a Au NP array with (a) 200 nm, (b) 600 nm, and (c) 2000 nm pitch.

To translate these results to practical light conversion efficiencies, we convert our results into an external quantum efficiency (EQE), intended as incident photon-to-product quantum yield
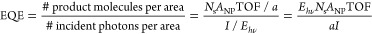
17where *N*_s_ is the number of active sites per Au surface area (10^19^ sites m^–2^), *A*_NP_ is the surface area per Au nanoparticle (approximated by 4π*r*^2^), *a* is the array unit cell area, and *E*_*h*ν_ is the energy of a 532 nm photon. It can be immediately seen (Figure 6) that the highest EQEs are obtained for the dense array and the lowest for the sparse array. This result is an immediate consequence of the high and low light absorption of the arrays (50 vs 0.5%). Furthermore, the overall maximum EQE is 24× higher for the 200 nm array than for any CW illumination intensity. Overall, for the two-dimensional Au NP arrays on glass we studied here, the highest EQEs were obtained for closely packed arrays (200 nm pitch) that were driven close to their melting point, below 100 kHz excitation and below 20 W/cm^2^. Finally, we also observed that the maximum internal quantum efficiency (IQE), intended as the activity per incident absorbed photon, is the same for all of the three array pitches (Figure S3). In other words, the maximum efficiency per active site is not affected by the pitch, but the total efficiency of the entire array is.

These observations lead to an important trade-off in reactor design. On the one hand, to reach maximum production with pulsed heterogeneous catalysis, sparse arrays must be used to limit collective heating and reach the high-intensity and high-repetition-rate region, where the highest TOF is achieved. On the other hand, sparse arrays also absorb only few incoming photons, thereby greatly decreasing the external quantum yield (Figure 6c). To increase the external quantum yield, the effective absorption cross section of the nanoparticles could be enhanced via nanophotonic approaches that enhance the incoming light field at the nanoparticle.^41−43^ Although this would shift the high-production region in Figure 5 to lower intensities and enhance the EQE of the array, it would also increase self-heating and collective heating by the same amount (eqs 1, 2, and 4). Thus, such an approach does not allow escaping the negative effects of collective heating.

It is, in principle, possible to reach high EQE and high TOF simultaneously. A potential solution may be to use three-dimensional (3D) reactor configurations, in which sparse arrays are smartly stacked, to achieve full absorption while also driving catalysis at maximum efficiency and activity. Alternatively, it may be possible to thermally engineer the heat transport properties of the catalyst support material to allow slow cooldown directly after excitation, to prolong catalytic activity for ns periods, while also allowing complete cooldown to ambient temperature in between pulses. This could, for instance, be achieved by exchanging the glass support for thermally conductive Al_2_O_3_ with a thin top-coating of thermally insulating SiO_2_.

A further important consideration to boost the EQE is the catalytic surface area (*A*_NP_). Although we here consider 50 nm Au particles with a poor surface-to-volume ratio (but a large absorption cross section), the benefits of a pulsed photothermal approach can be extended to antenna–reactor complexes, where the surface of the Au NP antenna is decorated with numerous smaller cocatalyst reactors.^44,45^ In these hierarchical structures, the plasmonic antenna provides the ultrafast localized heating, while the reactor cocatalysts offer suitable active sites for the catalytic reactions.

## Activation Energy

**Figure 7:**
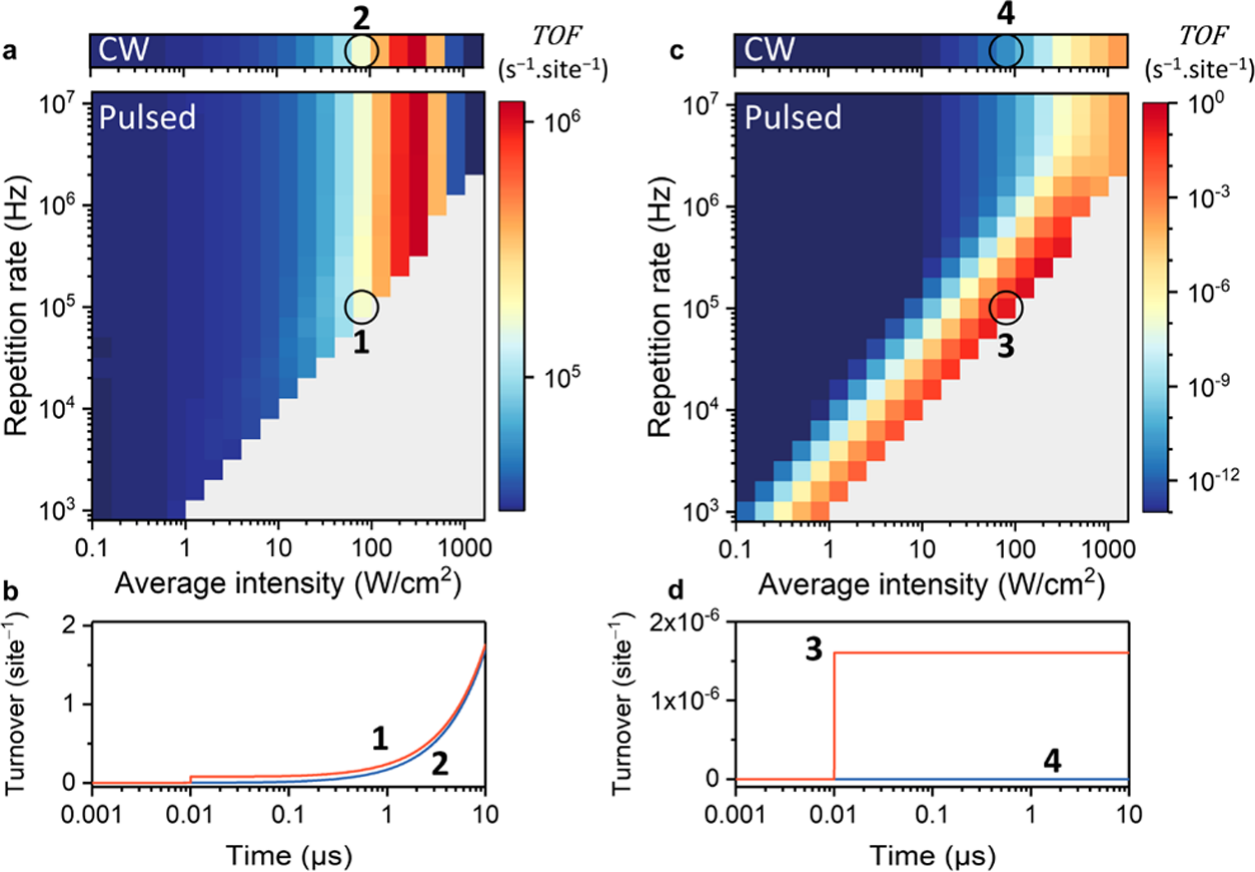
Role of activation energy in pulsed photothermal catalysis. (a, c) Reaction TOF under CW illumination (top) and under pulsed illumination (bottom) as a function of average light intensity and pulse repetition rate, for a Au NP array with 600 nm pitch and *E*_a_ = 50 kJ/mol (a) or 150 kJ/mol (c). (b, d) Catalytic turnover during a single pulse for 80 W/cm^2^ and 100 kHz pulsed illumination (orange curves) for *E*_a_ = 50 kJ/mol (b) or 150 kJ/mol (d). The optical pulse is centered at 10 ns. The blue traces show the turnover under CW illumination at the same intensity.

The reaction activation energy (*E*_a_) determines the temperature dependence of the reaction rate (Figure S4). It is therefore interesting to explore the role of activation energy in pulsed photothermal catalysis. To this end, the simulations for 600 nm pitch and *E*_a_ = 100 kJ/mol (Figure 4) were repeated with 50 and 150 kJ/mol activation energies (Figure 7). For an activation energy of 50 kJ/mol, pulsed illumination offers a negligible benefit over CW excitation for any combination of intensity and repetition rate (Figure 7a). For example, at 100 kHz and 80 W/cm^2^ (Figure 7, marks 1 and 2), pulsed illumination produces only 3% more than CW. This low productivity enhancement compared to the previous results is an immediate consequence of the fact that temperature affects the reaction rate less significantly for *E*_a_ = 50 kJ/mol than for *E*_a_ = 100 kJ/mol (Fig. S4). The enhanced rate during heat pulses is therefore less extreme compared to the rate in the dark interpulse period. Thus, even though CW illumination heats up only mildly (0.35 K per W/cm^2^), the much longer duration leads to a comparable TOF. This can be seen in Figure 7b, where the stepwise production during pulses is negligible compared to the slow production in the 10 μs pulse-to-pulse period.

The situation is reversed for *E*_a_ = 150 kJ/mol, where pulsed illumination has a much greater impact than for 100 kJ/mol (Figure 7c). For example, at 100 kHz and 80 W/cm^2^ (Figure 7, marks 3 and 4), pulsed illumination achieves a 10^10^ higher TOF than for CW illumination at the same intensity. Overall, these data demonstrate that pulsed photothermal heating offers no advantages for low activation energy reactions but can accelerate difficult catalytic processes with high activation energy barriers by orders of magnitude. Industrially relevant examples of such processes are CO/NO desorption on Ru, Rh, Pt, or Pd (100–150 kJ/mol);^1,46^ CH_4_ dissociation on Ni (90–105 kJ/mol);^47,48^ and CO oxidation on Pt or Ir (100 and 130 kJ/mol, respectively).^46^

## Reagent Poisoning Scenario

So far, we only considered the catalytic conversion of two chemical species A and B with the same adsorption energy (*E*_ads_^A^ = *E*_ads_^B^ = −50 kJ/mol). An important implication is that, in such a scenario, A and B do not compete for adsorption sites, that is, [A*] = [B*] for any temperature and any time. However, it is rarely the case that reagents have the same binding energy and surface coverage. Especially for large differences in binding energy, one reagent dominates surface coverage, up to the limit where it poisons the catalyst surface and prevents binding of other reagents. In this section, we demonstrate that, under such reagent poisoning scenario (Figure 3, scenario 2), pulsed illumination provides access to surface coverages and, therefore, reaction rates that are normally inaccessible under steady-state conditions.

**Figure 8:**
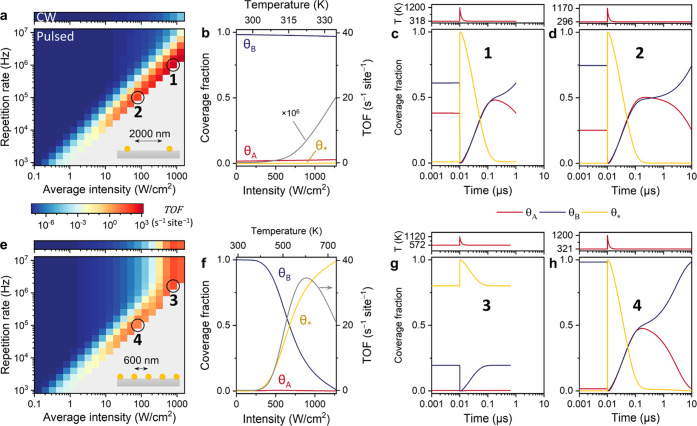
Microkinetic modeling for the scenario of reagent poisoning (scenario 2 in Figure 3) for an Au NP array with 2000 nm pitch (a–d) and 600 nm pitch (e–h). (a, e) Reaction TOF as a function of light intensity and pulse repetition rate. (b, f) Steady-state coverage fractions of A*, B*, empty sites, and the reaction TOF (gray, right axis) as a function of C.W. intensity (bottom axis) and temperature (top axis). (c, d, g, h) Nanoparticle surface temperature (top panels) and coverage fractions (bottom panels) of A*, B*, and empty sites during a single pulse for the illumination parameters and pitch indicated by the numbered circles in panels (a) and (e): (c) 803 W/cm^2^, 1 MHz, and 2000 nm pitch; (d) 80 W/cm^2^, 100 kHz, and 2000 nm pitch; (g) 803 W/cm^2^, 1.6 MHz, and 600 nm pitch; (h) 80 W/cm^2^, 100 kHz, and 600 nm pitch. The optical pulse is centered at 10 ns.

We simulated a potential energy landscape with a large difference in binding energy between A and B (*E*_ads_^A^ = −50 kJ/mol, *E*_ads_^B^ = −70 kJ/mol). The pitch of the array is initially chosen to be 2000 nm to exclude collective heating and the activation energy set to 100 kJ/mol (Figure 8). For CW illumination (Figure 8a, top panel, and Figure 8b), the catalyst activity is severely reduced with respect to the equal binding energy scenario (Figure 5b) because B poisons the catalyst surface ([B*] ≥ 0.97). In stark contrast, for pulsed illumination along the high peak temperature diagonal, the catalyst activity is very high, up to 10^9^× higher than for CW illumination. By inspecting the time-dependent surface coverages, it becomes clear that poisoning does not occur for such conditions (Figure 8c,d). For instance, at 1 MHz and 803 W/cm^2^ (mark 1, Figure 8c), the high-temperature pulse fully depletes the surface through reaction or reagent desorption. In the next 1 μs, the surface is cold and refills with reagents in an approximately 1:1 ratio according to their (almost equal) adsorption rate. Crucially, during this period, θ_A_ and θ_B_ start to slowly approach their steady-state values (Figure 8b) but are still far from equilibrium values when the next pulse arrives. In other words, the time required for arriving at the steady state is longer than the pulse-to-pulse time, which is approximately 100 μs at the array’s temperature of 319 K (Fig. S5).

The important consequence is that θ_A_ and θ_B_ at the start of the pulse have values that are unlike any concentration under CW, and chemistry is therefore driven at a much higher rate, according to eq 12. Such non-steady-state surface coverages are also present for lower repetition rates, such as 100 kHz and 80 W/cm^2^ (mark 2, Figure 8d), until the pulse-to-pulse time approaches 100 μs. Taken altogether, these results show that a completely different reaction regime can be attained, in which the reactant concentrations are dictated by adsorption kinetics during the low-temperature stage, while catalytic rates are dominated by the transient high temperatures provided by the electromagnetic pulses. Such a kinetic regime is entirely beyond the reach of conventional, steady-state thermal catalysis.

Because the time required for arriving at the steady-state coverages is very temperature-sensitive (Figure S5) due to the temperature dependence of adsorption and desorption processes, collective heating strongly affects whether non-steady-state surface coverages can be accessed. To demonstrate this effect, the simulations were also carried out for 600 nm pitch, for which large collective heating contributions are expected (Figure 8e–h). In the limit of significant collective heating (e.g., at 1 MHz and 803 W/cm^2^ intensity, mark 3, Figure 8g), surface coverages arrive at their steady-state values well within the pulse-to-pulse period. Thus, pulsed catalysis occurs with the same “poisoned” surface coverages as for steady-state heating, and both CW and pulsed illumination result in the same, limited TOF. Also, at lower intensities (e.g., at 100 kHz and 80 W/cm^2^, mark 4, Figure 8h), collective heating drives the surface coverages toward steady-state concentrations. Thus, yet again, the array’s pitch and the amount of collective heat play an important role in the efficacy of pulsed catalysis. Non-steady-state, nonpoisoned surface coverages with substantially elevated reaction rates can only be achieved if the nanoparticles are kept cool in between pulses and reagent redistribution is prevented.

Finally, we note that it is easy to imagine scenarios where dynamics of surface refilling and redistribution are much slower due to lower adsorption and desorption rates (i.e., lower sticking coefficients), the presence of adsorption and desorption activation barriers, or lower Arrhenius pre-exponential factors. Although this vast parameter space is out of the scope of this work, we can offer some general predictions. For low adsorption rate scenarios, fast pulsing is likely to be counterproductive, as surface coverages are still low when the next pulse arrives. For low-desorption-rate scenarios, it will take longer to arrive at steady-state conditions, and pulsed catalysis may exploit non-steady-state coverages up to a higher degree of collective heating.

In general, we predict that there will be an optimum repetition rate that resonates with the kinetics of adsorption. This fact calls for targeted experiments in which the reaction rate is measured while changing the repetition rate of pulsed illumination over several orders of magnitude.

## Product Poisoning Scenario

**Figure 9:**
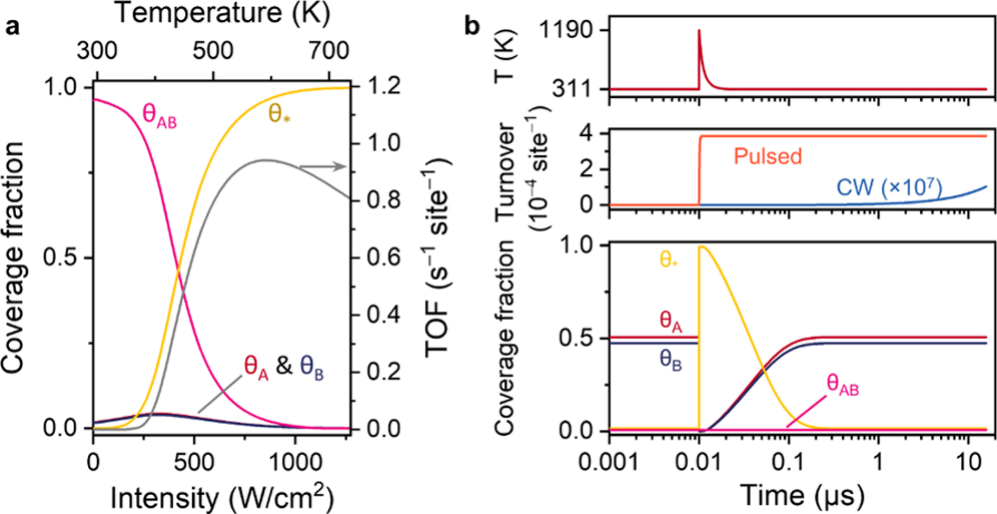
Microkinetic modeling for the scenario of product poisoning (scenario 3 in Figure 3) for an Au NP array with 600 nm pitch. (a) Steady-state coverage fractions of A*, B*, AB*, and empty sites, and the reaction TOF (right axis) as a function of CW illumination intensity (bottom axis) and temperature (top axis). (b) Nanoparticle surface temperature (top), reaction turnover (orange, middle), and coverage fractions (bottom) of A*, B*, AB*, and empty sites, during a single pulse for 51 W/cm^2^ and 63 kHz pulsed illumination. The optical pulse is centered at 10 ns. The reaction turnover under 51 W/cm^2^ CW illumination is shown in blue (middle panel).

A further, important scenario we considered is the strong, inhibitive binding of the product or catalyst poisoning by the product (Figure 3, scenario 3). In this scenario, the high surface occupation by the product prevents coverage by the reagents and therefore inhibits the catalytic rate. We predicted that a strong heat pulse would eliminate the poison and greatly improve the catalytic rate compared to CW illumination. For this scenario, we modified the energetic landscape by placing the product in an energy well, where the desorption is slowed by a high binding enthalpy (*E*_ads_^AB^ = −120 kJ/mol) and the introduction of a large reaction enthalpy change (Δ*H*_rxn_ = −50 kJ/mol) prevents the back reaction (Figure 3). Under steady-state CW excitation of the Au nanoparticle array (600 nm pitch) and moderate intensity (<250 W/cm^2^), the product (AB) indeed occupies 90–95% of the catalyst and severely inhibits the reaction rate (Figure 9a). This inhibitory effect is removed in the high-intensity regime (>250 W/cm^2^), and the catalyst achieves an activity close to the scenario without product inhibition (compare Figures 4d and 9a).

For this product poisoning scenario, mapping out the entire intensity and FHWM parameter space proved to be too resource intensive because, for a fair comparison with CW heating, the catalyst must first reach a “pseudo-steady state” in which the surface coverage of the poison (θ_AB_) does not change during a single pulse period. In other words, the poison AB must first be produced, up to the point where the rates of production and desorption of AB during the pulse are equal. We therefore focused on a few combinations of irradiation intensity and repetition rate along the high-temperature diagonal (Figure 4a) and executed 2000 prepulses to reach the pseudo-steady state.

In the moderate-intensity regime (e.g., 51 W/cm^2^ at 63 kHz, Figure 9b), the high-temperature pulse successfully prevents a buildup of products (θ_AB_ < 1%) and forces them to desorb. The catalytic turnover is therefore roughly 3 × 10^7^ times higher than for CW conditions. Notably, the surface coverages of the reagents (θ_A_ and θ_B_) are more than 10× higher than any surface coverage under steady-state heating. Results with 202 W/cm^2^ and 250 kHz excitation were very similar (Figure S6). However, at a high intensity (803 W/cm^2^ and 1.6 MHz), pulsed and CW illumination both led to a negligible turnover because of the extremely low surface coverages (Figures S6 and 4). Overall, these results demonstrated that illumination conditions exist for which catalysis under pulsed excitation is unencumbered by product poisoning and is characterized by surface coverages that are inaccessible under steady-state heating.

## Application of Pulsed Microkinetic Modeling to CO Oxidation

Finally, we also applied the pulsed microkinetic model to CO oxidation on a Pt catalyst, for which the kinetic parameters and energetic landscape were taken from literature reports (parameters and rate equations reported in the Supporting Information).^4,46,49^ For this scenario, we consider a plasmonic Au–Pt core–shell nanoparticle array (600 nm pitch) with an identical absorptivity^50^ and identical thermal properties to the 50 nm diameter Au NPs used in the rest of this work. Further, to acknowledge that CO coverage directly affects CO binding energy and reaction activation energy through neighbor effects,^46^ the desorption energy of CO is taken to decrease linearly from 146 to 84 kJ/mol and the activation energy to decrease linearly from 101 to 51 kJ/mol, both as a function of θ_CO_.^49^ Finally, the binding of oxygen is very strong (160 kJ/mol), while the binding of CO_2_ is weak (19 kJ/mol).^4^

**Figure 10:**
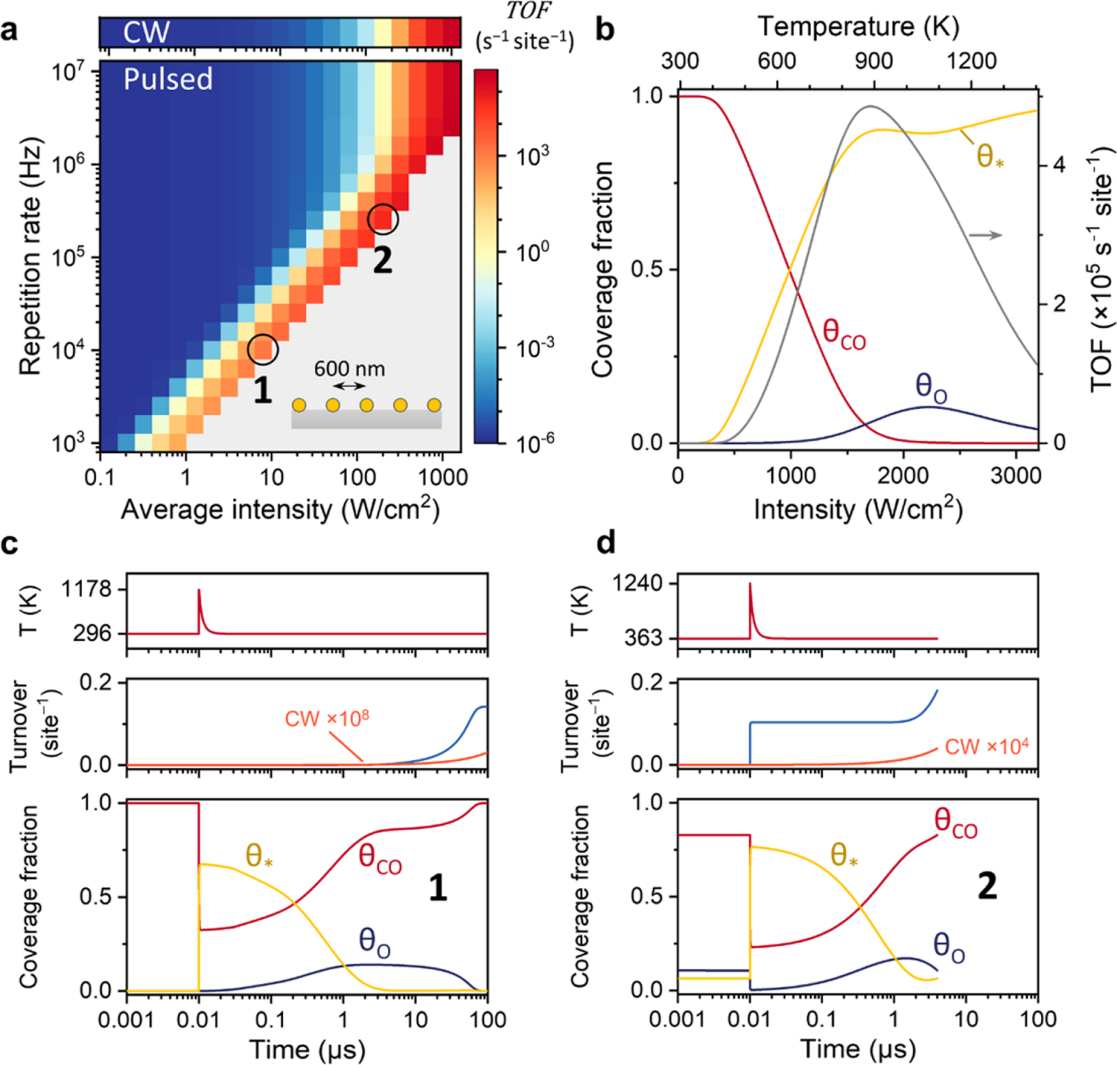
Microkinetic modeling of CO oxidation under CW and pulsed illumination for an array of Au–Pt core–shell NPs with 600 nm pitch. (a) Reaction TOF (CO_2_ molecules site^–1^ s^–1^) under CW illumination (top) and under pulsed illumination (bottom). The pulse conditions labeled with 1 and 2 correspond to the blue turnover data in panels (c) and (d). (b) Steady-state coverage fractions of CO*, O*, and empty sites, and the reaction TOF (gray, right axis) as a function of CW illumination intensity (bottom axis) or temperature (top axis). (c, d) Nanoparticle surface temperature (top), reaction turnover (middle), and coverage fractions of CO*, O*, and empty sites (bottom) for a single pulse period with an intensity of 8 W/cm^2^ and a repetition rate of 10 kHz (c) and for an intensity of 200 W/cm^2^ and a repetition rate of 250 kHz (d). In both cases, the optical pulses are centered at 10 ns. The reaction turnover with the same intensity CW illumination is shown in orange.

Again, we directly compared the TOF (CO_2_ molecules produced site^–1^ s^–1^) between CW and pulsed illumination (Figure 10). Under steady-state heating (Figure 10a, upper panel, and Figure 10b), the surface is fully poisoned by CO below 450 K, thereby restricting binding of oxygen and CO oxidation. In contrast, for pulsed illumination (Figure 10a), we find pulse conditions up to ±200 W/cm^2^ intensity for which the reaction rate is orders of magnitude higher than that at CW illumination, at the same intensity.

To elucidate the origin of this increased reactivity, we examine the surface coverages of CO and O and the reaction turnover during a single pulse (Figure 10c,d). For 10 KHz and 8 W/cm^2^ illumination (Figure 10c), the heat pulse itself produces a negligible amount of CO_2_ due to the full coverage by CO, but it does cause partial desorption of CO. In the following 100 μs, oxygen is allowed to adsorb to the surface and the oxidation takes place, facilitated by a lower *E*_a_ at high θ_CO_, until the surface is poisoned once more, as a result of the consumption of O and the stronger sticking coefficient of CO. These results are in line with the work of Niemantsverdriet et al., who have experimentally shown for electrically heat-pulsed CO oxidation on Pt (25 μs pulse width, 20 Hz) that “depoisoning” of the surface upon heat-pulsing leads to a 40× higher TOF at the same energy consumption. For 250 KHz and 200 W/cm^2^ illumination (Figure 10d), half of the CO_2_ is produced directly after the pulse, and the other half is produced in the following 4 μs. During the pulse, the surface is partially occupied by O with a high O–CO ratio that cannot be found at any steady-state temperature, and hence, the CO–O oxidation reaction takes place efficiently during the pulse. In other words, a repetition rate of 250 kHz resonates better with the dynamics of CO and O coverages than 1 kHz at these particular reactor conditions.

Taken together, the results of this CO oxidation case study, based on realistic literature parameters, demonstrate once more that pulsed photothermal catalysis offers the possibility to control surface coverages, desorb catalyst poisons from the surface, and can result in a higher energy efficiency.

## Recommendations for Experimental Realization

For the longest time, the realization of pulsed operation of catalysts has been out of reach because of the limited temporal control over the reactor heating and cooling. With pulsed plasmonic heating and the advances in pulsed light sources, we now have the tools available to achieve catalyst heating and cooling on the same timescales as catalytic processes. In this work, we have shown that, even with an unoptimized catalyst array, under pulsed illumination, we can reach reaction rates hundreds or thousands of times higher than under CW illumination with the same energy input. In contrast, experimental results with CO oxidation and CO_2_ hydrogenation with either electrical or optical pulses have so far demonstrated conversion increases of only 40× to 50×.^4,5,12^ Thus, there is still a vast and exciting parameter space to explore to tune catalyst activity, selectivity, and energy efficiency in real-world catalysis. Our calculations lead to the following recommendations toward experimental realization:

- **Work close to the photoablation limit**. Pulsed light can result in drastically higher catalytic productivity and energy efficiency but only when particles are driven to temperatures nearby their photoablation limit. Thus, short (<50 ps) and intense light pulses are required, and the expected peak temperatures must be calculated from pulse energy density and absorption cross sections (eq 2). To prevent photoablation, the catalyst photostability must be explored by systematically increasing the pulse energy density while confirming nanoparticle integrity.
- **Limit collective heating**. Whereas for CW illumination collective heating is highly favorable, for pulsed catalysis, it can be a limiting factor due to reduced surface coverages and a smaller operational window of pulse energy before the material starts to photoablate. Thus, plasmonic nanoparticle arrays are best driven in a regime with low collective heating effects to allow for high productivity per pulse and a favorable combination of high EQE and TOF. Thermal management will depend dramatically on the optical absorption (nanophotonic design) and thermal dissipation of the photocatalyst as well as on the thermal management within the reactor. In particular, experimental conditions should favor heat dissipation away from the catalytic surface to minimize temperature homogenization effects.
- **Match pulse parameters to chemical kinetics**. Our results highlight that choosing the right conditions is decisive for exploiting pulsed illumination in heterogeneous catalysis. Under the right conditions, substantially higher TOF and energy efficiencies can be achieved. The ideal conditions will resonate with the chemical kinetics of the catalytic system (adsorption and desorption equilibria, surface coverages, reaction rates, mass transport, etc.). To find these conditions experimentally, the repetition rate must be varied while keeping the pulse energy density constant (at a value nearby the catalyst melting point). To assist this search, microkinetic simulations, such as those demonstrated in this work, can narrow down the pulse parameter space for specific catalyst systems.
- **Mind material stability**. Conventional, plasmonic metals such as gold and silver melt, deform, and lose function under intense pulsed illumination, even well before their bulk melting point.^39,40^ Thus, novel robust plasmonic nanomaterials must be developed that can provide access to the high-temperature region where pulsed catalysis becomes most effective. Examples of such materials are thermally robust metal nitrides (e.g., TiN and HfN)^51,52^ and metal carbides (e.g., HfTa_4_C_5_).^53^ Further functionalization of these materials with catalytic metals, such as single-atoms catalysts,^54,55^ could lead to robust photothermal catalysts, especially tailored for pulsed light.

## Conclusions and Outlook

We have explored the use of pulsed light excitation for photothermal heterogeneous catalysis using heat dissipation and time-dependent microkinetic modeling. Through a number of model kinetic scenarios, as well as a realistic scenario of Pt-catalyzed CO oxidation, we have highlighted how the use of pulsed light results in a favorable, non-steady-state mode of operation that offers several distinct advantages: higher energy efficiency, higher turnover per site than for any steady-state temperature, operation at room temperature, robustness against catalyst poisons, and access to surface coverages of reagents that are markedly different from steady-state operation. Since reagent surface coverages are intimately related to reaction selectivity, we also predict that pulsed light can potentially result in control over the product distribution in more complex reactions, such as CO_2_ hydrogenation,^56^ Fischer–Tropsch synthesis,^57^ or CH_4_ pyrolysis.^6^

However, we also consistently emphasize that all of these benefits are highly situational and strongly depend on the pulse width, pulse energy density, interparticle distance, and the potential energy landscape of the reaction. Perhaps unexpectedly, whereas collective heating is highly favorable for CW excitation, it is detrimental for pulsed excitation: intense collective heating prevents the surface to become fully covered in between pulses, while also speeding up the redistribution of reagent coverages toward unfavorable steady-state coverages. Concretely, for the nonexhaustive set of physical and kinetic parameters we have focused on, pulsed heating results in an enhanced performance compared to steady-state heating when the system (i) is optically excited with a pulse of <50 ps, (ii) is operated at peak temperatures close to the melting point of the photothermal catalyst, (iii) has a large interparticle distance to limit collective heating, and (iv) has a reaction activation energy of at least 100 kJ/mol.

Finally, it must be emphasized that the simplicity of our model naturally leads to generic conclusions. While we identify clear trends and benefits to pulsed photothermal catalysis, the unique kinetic landscape of each catalyst system may lead to large deviations from our predictions. For example, our model assumes simple catalytic mechanisms and temperature-independent pre-exponential factors, does not consider potential limitations due to mass transport at high rates, does not consider the contribution of reactor geometry, and neglects the surface coverage dependence of the adsorption energy and activation barrier (except for our case study of CO oxidation). In experimental realizations, specific cases must be individually investigated, ideally under a wide range of conditions. The exploration of such vast parameter space is now enabled by the recent impressive innovations of (ultra)fast affordable light sources with tunable power, operational up-time, pulse width, and repetition rate. Additional knobs to turn are pulse-to-pulse energy and delay variations, which could unlock dynamic control over thermal chemistry at the pulse-to-pulse level. Experimental exploration of this vast parameter space will no doubt uncover new catalytic phenomena.

Overall, our work enables the rational design and interpretation of the necessary experiments to verify the potential of pulsed photothermal catalysis, which may lead to a greener, more sustainable, and more process-intensive operation of heterogeneous catalysis.

## Supporting Information Available

Details on microkinetic modeling; finite element method heat-transfer simulations; and microkinetic simulation results (PDF)
