# Supplementary material for: Pulsed Photothermal Heterogeneous Catalysis
Source: ACS Catal. 2023 Feb 22;13(5):3419–32. doi: 10.1021/acscatal.2c05435 (PMC9990069; doi:10.1021/acscatal.2c05435)
Supplement: Supplementary file 1 — cs2c05435_si_001.pdf [file cs2c05435_si_001.pdf]

Pulsed Photothermal Heterogeneous Catalysis

Andrea Baldi and Sven H. C. Askes.
Department of Physics and Astronomy, Vrije Universiteit Amsterdam
s.h.c.askes@vu.nl / a.baldi@vu.nl

SUPPORTING INFORMATION

Microkinetic modelling

The system of ordinary differential equations (ODEs) governing the microkinetic model (Equation S1 to Equation S5), where each of the rate constants was time dependent, was numerically integrated using a time-dependent ODE solver implemented in COMSOL software. Specifically, a time-dependent implicit solver was used according to the backward-differential formulation method (BDF) with an intermediate time-stepping and a relative tolerance of 1×10^{-9}. The light pulse was centered at 10 ns. To accurately capture the catalytic dynamics around the ultrafast pulse, the model was solved with smaller time steps around the pulse. Each light pulse was discretized as follows:

- from 0 to 9 ns (before the pulse) with 1 ns step intervals
- from 9 to 11 ns (during the pulse) with 0.005 ns step intervals
- from 11 to 30 ns (right after the pulse) with 0.1 ns step intervals
- from 30 ns to the next pulse with 1000 step intervals

The simulations for scenarios 1 and 2 started with an empty catalyst surface ([*] = 1; [A*] = [B*] = [AB*] = 0). A total of 21 pulses were simulated per illumination condition (intensity and repetition rate), of which the first 20 pulses "pre-conditioned" the catalyst to attain a pseudo-steady state. In this pseudo-steady state, the surface coverages at the start of the pulse-window ($t = (i - 1)/f$ for the ith pulse) are identical to the ones at the end of the pulse-window ($t = i/f$), as shown for instance in the bottom panel of Fig. 4e in the main text. Ultimately, only the data of the 21st pulse was used as final result for further data processing. As discussed in the section "Product poisoning scenario" in the main text, the simulations for scenario 3 were performed with 2000 pre-conditioning pulses, where the results of the 2001st were used as final data set. The turnover frequency (TOF, in units of product molecules per site, per second) was calculated by integrating Equation S4 for a single pulse period and multiplying by the repetition rate (Eq. 16 in the main text).

$$\frac{dA^*}{dt} = k_{ads}^{A}[*] - k_{des}^{A^*}[A^*] - k_{rxn}[A^*][B^*] \qquad \text{Equation S1}$$

$$\frac{dB^*}{dt} = k_{ads}^{B}[*] - k_{des}^{B^*}[B^*] - k_{rxn}[A^*][B^*] \qquad \text{Equation S2}$$

$$\frac{dAB^*}{dt} = k_{rxn}[A^*][B^*] - k_{des}^{AB^*}[AB^*] \qquad \text{Equation S3}$$

$$\frac{dAB}{dt} = k_{des}^{AB^*}[AB^*] \qquad \text{Equation S4}$$

$$\frac{d*}{dt} = -k_{ads}^{A}[*] + k_{des}^{A^*}[A^*] - k_{ads}^{B}[*] + k_{des}^{B^*}[B^*] + k_{rxn}[A^*][B^*] + k_{des}^{AB^*}[AB^*] \qquad \text{Equation S5}$$

Microkinetic modelling of CO oxidation on Au-Pt core-shell nanoparticle arrays

The microkinetic model was adjusted for the case of CO oxidation by considering the following surface reactions according to a Langmuir-Hinshelwood mechanism:

CO adsorption	$CO + * \xrightarrow{k_{ads}^{CO}} CO^*$	Equation S6
CO desorption	$CO^* \xrightarrow{k_{des}^{CO}} CO + *$	Equation S7
Dissociative O_2 adsorption	$O_2 + 2* \xrightarrow{k_{ads}^{O_2}} 2O^*$	Equation S8
Associative O_2 desorption	$2O^* \xrightarrow{k_{des}^{O_2}} O_2 + 2*$	Equation S9
CO oxidation (forward reaction)	$CO^* + O^* \xrightarrow{k_{rxn}} CO_2^* + *$	Equation S10
CO_2 dissociation (back-reaction)	$CO_2^* + * \xrightarrow{k_b} CO^* + O^*$	Equation S11
CO_2 desorption	$CO_2^* \xrightarrow{k_{des}^{CO_2}} CO_2 + *$	Equation S12

Which together give rise to the following rate equations for the coverage fractions of CO*, O*, CO_2*, free sites (*), and the produced CO_2:

$$\frac{dCO^*}{dt} = k_{ads}^{CO}[*] - k_{des}^{CO}[CO^*] - k_{rxn}[CO^*][O^*] + k_b[CO_2^*][*] \quad \text{Equation S13}$$

$$\frac{dO^*}{dt} = 2k_{ads}^{O_2}[*]^2 - 2k_{des}^{O_2}[O^*]^2 - k_{rxn}[CO^*][O^*] + k_b[CO_2^*][*] \quad \text{Equation S14}$$

$$\frac{dCO_2^*}{dt} = k_{rxn}[CO^*][O^*] - k_b[CO_2^*][*] - k_{des}^{CO_2}[CO_2^*] \quad \text{Equation S15}$$

$$\frac{dCO_2}{dt} = k_{des}^{CO_2}[CO_2^*] \quad \text{Equation S16}$$

$$\frac{d*}{dt} = -k_{ads}^{CO}[*] + k_{des}^{CO}[CO^*] - 2k_{ads}^{O_2}[*]^2 + 2k_{des}^{O_2}[O^*]^2 + k_{rxn}[CO^*][O^*] - k_b[CO_2^*][*] + k_{des}^{CO_2}[CO_2^*] \quad \text{Equation S17}$$

$$k_{des}^{CO} = A_{des}^{CO} e^{-\frac{(E_{des}^{CO} - B[CO])}{RT}} \quad \text{Equation S18}$$

$$k_{rxn} = A_{rxn} e^{-\frac{(E_a - C[CO])}{RT}} \quad \text{Equation S19}$$

As CO-oxidation catalyst we consider Au-Pt core-shell nanoparticle arrays with a thin Pt shell (50 nm total diameter), where the absorption cross section can be approximated by that of Au NPs (σ_{abs} = 2 × 10^{-14} m^2, see main text).[1] Thus, the heating and cooling dynamics of these nanoparticle arrays were identical to those described for Au NPs. Representative energetic and kinetic parameters were taken from literature on CO oxidation on Pt,[2,3] with A_{rxn} set to 1.65 × 10^{14}, A_{des}^{CO} to 1.25 × 10^{15}, and the pre-exponential factor of the other rate constants set to 1 × 10^{14}. The sticking coefficients of CO and O_2 were set to 0.84 and 0.02, respectively, and the pressures of CO and O_2 were 0.005 and 0.095 atm, without considering mass transport in the reactor. The desorption energy of CO was set to 146 kJ/mol (1.51 eV), the desorption energy of O was set to 160 kJ/mol (1.66 eV), the reaction activation energy was set to 101 kJ/mol (1.045 eV), the energy change of the reaction was set to -41 kJ/mol (−0.43 eV), and the desorption energy of CO_2 was set to 19 kJ/mol (0.2 eV).[3] Further, because the required energy to desorb CO decreases with CO coverage, the energy dependence of the desorption rate was linearly modified by the CO coverage (Equation S18, where B was set to 62 kJ/mol), according to Rinnemo *et al.*[3] Finally, because the activation energy of CO oxidation is also dependent on the CO coverage, the energy dependence of the reaction rate was linearly modified by the CO coverage (Equation S19, where C was set to 50 kJ/mol).[4]

[1] Henglein, A. Preparation and Optical Absorption Spectra of $Au_{core}Pt_{shell}$ and $Pt_{core}Au_{shell}$ Colloidal Nanoparticles in Aqueous Solution. *J. Phys. Chem. B* **2000**, *104* (10), 2201–2203. https://doi.org/10.1021/jp994300i.

[2] Zhu, Z.; Weber, M.; Verheijen, M. A.; Bol, A. A.; Spinu, V.; Özkan, L.; Backx, A. C. P. M. (Ton); Niemantsverdriet, J. W. (Hans); Fredriksson, H. O. A. Novel Microreactor and Generic Model Catalyst Platform for the Study of Fast Temperature Pulsed Operation – CO Oxidation Rate Enhancement on Pt. *Chemical Engineering Journal* **2021**, *425*, 131559. https://doi.org/10.1016/j.cej.2021.131559.

[3] Rinnemo, M.; Kulginov, D.; Johansson, S.; Wong, K. L.; Zhdanov, V. P.; Kasemo, B. Catalytic Ignition in the COO2 Reaction on Platinum: Experiment and Simulations. *Surface Science* **1997**, *376* (1), 297–309. https://doi.org/10.1016/S0039-6028(96)01572-5.

[4] Zhdanov, V. P. *Elementary Physicochemical Processes on Solid Surfaces*; Twigg, M. V., Spencer, M. S., Series Eds.; Fundamental and Applied Catalysis; Springer US: Boston, MA, 1991. https://doi.org/10.1007/978-1-4899-2373-8.

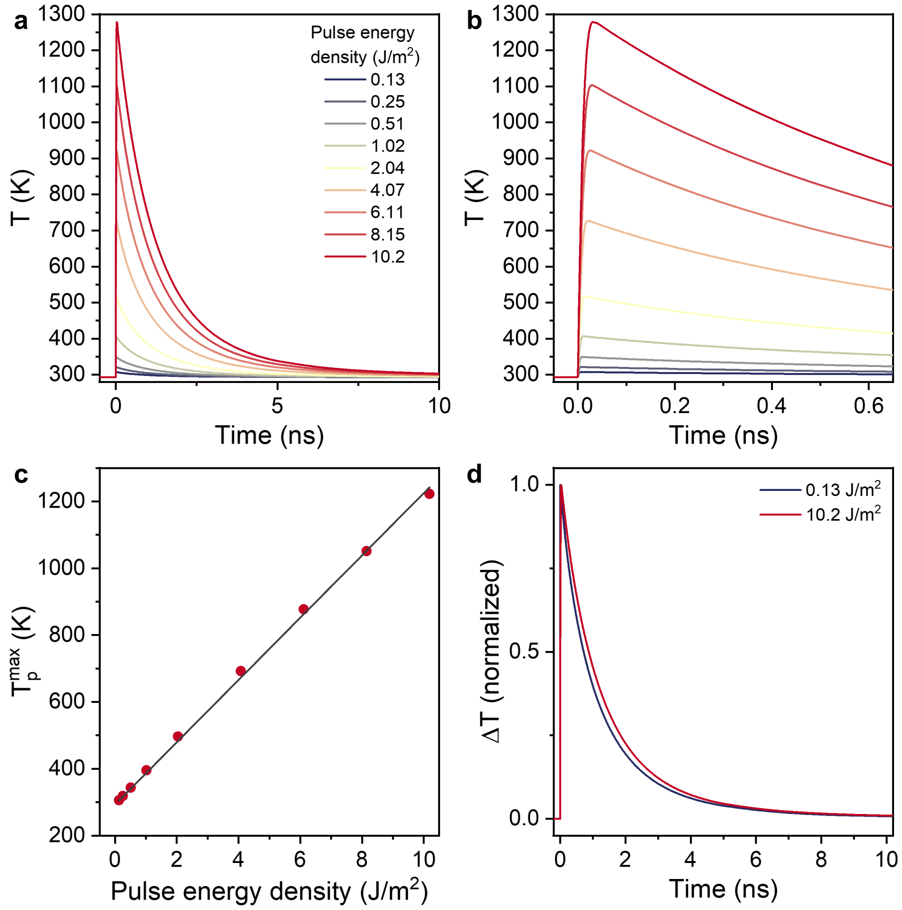

Fig. S1. Finite element method simulations of a 50 nm diameter Au nanosphere supported on SiO_2 in air, using a two-temperature model and 50 fs pulsed excitation at variable pulse energy density (0.13 – 10.2 J/m^2). a) Decay of the averaged surface temperature of the nanoparticle at variable pulse energy density. b) Same data as panel a, but zoomed in on the first 600 ps. c) Maximum averaged surface temperature as a function of pulse energy density (red data points) and linear fit to the data (black line). d) Normalized decay of the averaged surface temperature for 0.13 and 10.2 J/m^2. The data show that, despite non-linearities in thermal conductivity and heat capacity, the maximum temperature is a linear function of pulse energy density, while changes in lifetime are minimal and can therefore be neglected in the microkinetic modelling.

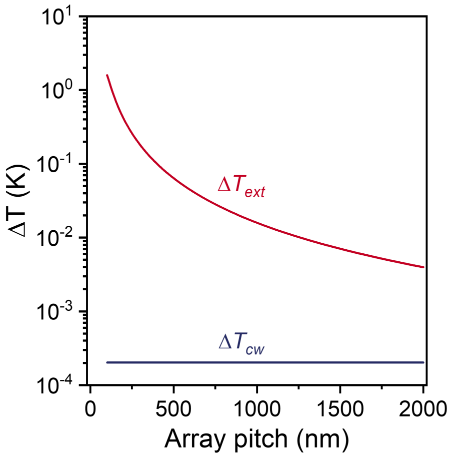

Fig. S2. Nanoparticle self-heating under CW conditions (blue) and collective heating of a nanoparticle array (red) as a function of array pitch, according to Eq. 1 and Eq. 4. Parameters used: $\sigma_{abs} = 2 \times 10^{-14}$ m^2, power = 1 mW, beam diameter = 1 mm, $\kappa_{effective}$ = 0.4 W/m.K, and r = 25 nm. This comparison shows that, even for sparse arrays, under the parameter ranges relevant to photothermal heterogeneous catalysis and explored in this work, collective heating is responsible for most of the temperature increase.

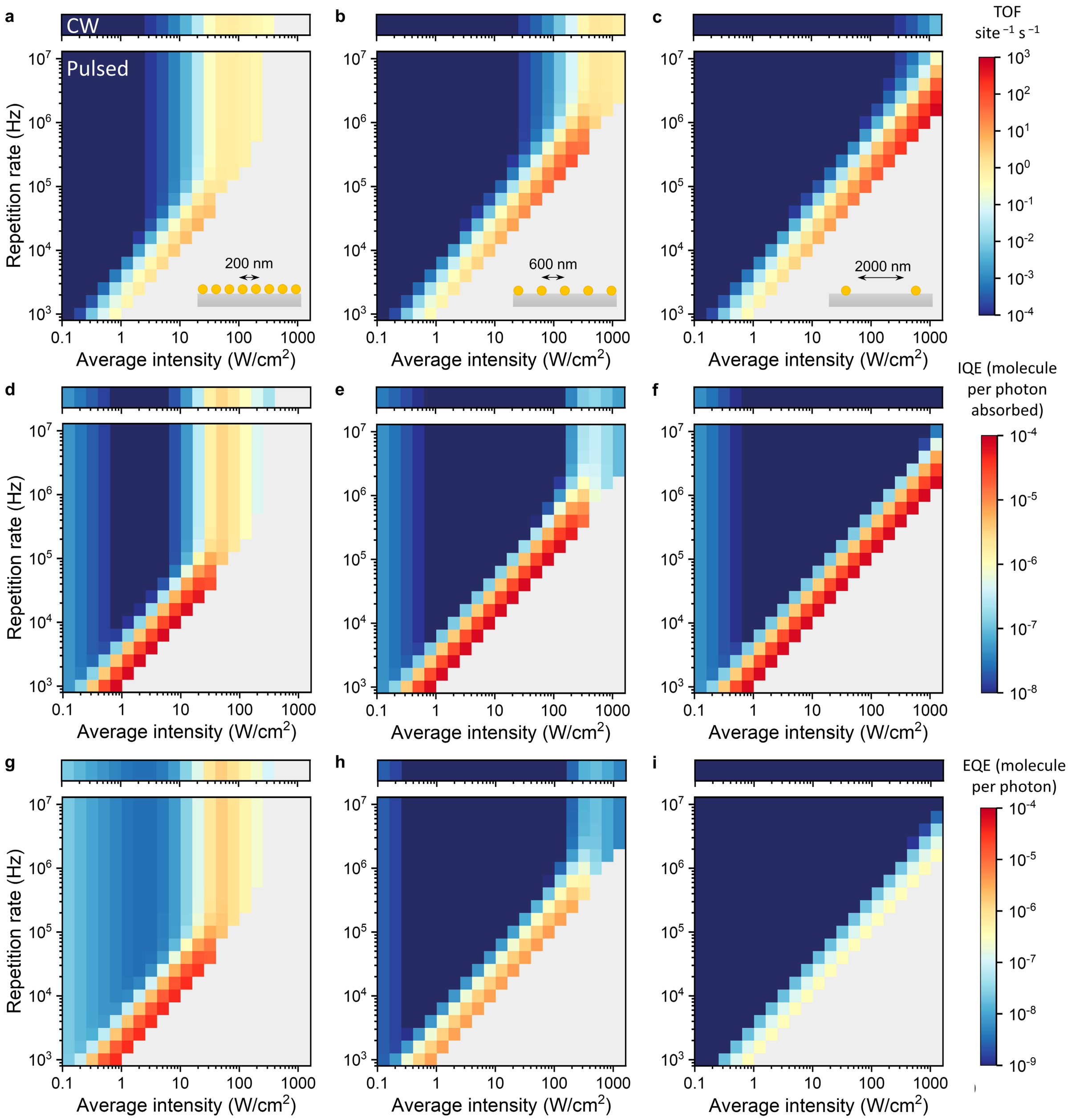

Fig. S3. Reaction TOF (top row), internal quantum efficiency (IQE, middle row, expressed as product molecules per photon absorbed), and external quantum efficiency (EQE, bottom row, expressed as product molecules per incident photon), as a function of light intensity and pulse repetition rate, for a Au NP array with 200 nm pitch (left column), 600 nm (middle column), and 2000 nm pitch (right column). The IQE is calculated by multiplying the EQE (Eq. 17) by the fraction of absorbed photons.

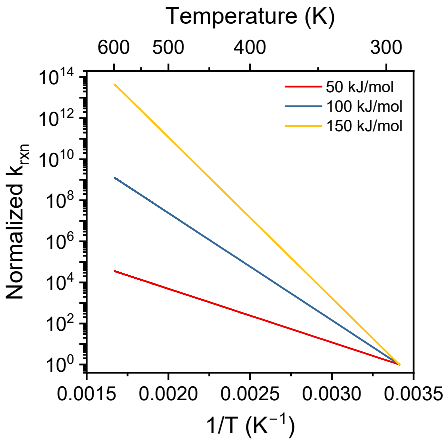

Fig. S4. Reaction rate constant, normalized to T = 293 K, according to the Arrhenius equation as a function of temperature for an activation energy of 50, 100, and 150 kJ/mol.

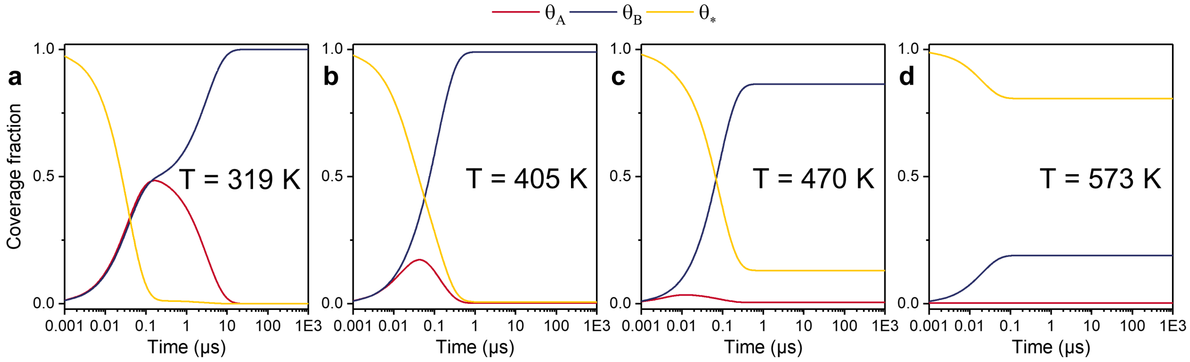

Fig. S5. Time-dependent coverages of A*, B*, and empty sites for Scenario 2 (H^A_{ads}= 50 kJ/mol, H^B_{ads} = 70 kJ/mol), at 319 K (a), 405 K (b), 470 K (c), and 573 K (d). The simulation starts with an empty surface. The data show that temperature dictates what non-steady state surface coverages of A* and B* are accessible.

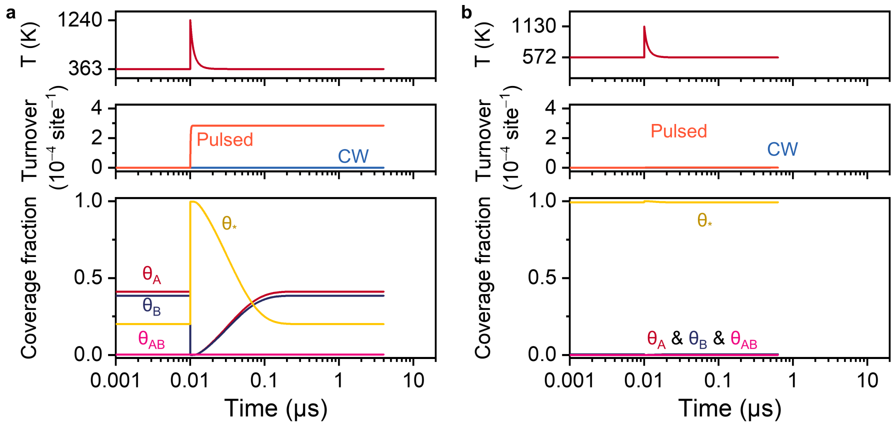

Fig. S6. Nanoparticle surface temperature (top panels), reaction turnover (orange, middle panels), and coverage fractions (bottom panels) of A*, B*, AB*, and empty sites, for a single optical pulse at 202 W/cm^2 & 250 kHz (a) and for 803 W/cm^2 & 1.6 MHz (b). The pulse is centered at 10 ns. The corresponding reaction turnovers under 202 W/cm^2 and 803 W/cm^2 CW illumination are given in blue (middle panels).
